# Entropic Stabilization of Proteins and Its Proteomic Consequences

**DOI:** 10.1371/journal.pcbi.0010047

**Published:** 2005-09-30

**Authors:** Igor N Berezovsky, William W Chen, Paul J Choi, Eugene I Shakhnovich

**Affiliations:** 1 Department of Chemistry and Chemical Biology, Harvard University, Cambridge, Massachusetts, United States of America; 2 Department of Biophysics, Harvard University, Cambridge, Massachusetts, United States of America; Buffalo Center of Excellence in Bioinformatics, United States of America

## Abstract

Evolutionary traces of thermophilic adaptation are manifest, on the whole-genome level, in compositional biases toward certain types of amino acids. However, it is sometimes difficult to discern their causes without a clear understanding of underlying physical mechanisms of thermal stabilization of proteins. For example, it is well-known that hyperthermophiles feature a greater proportion of charged residues, but, surprisingly, the excess of positively charged residues is almost entirely due to lysines but not arginines in the majority of hyperthermophilic genomes. All-atom simulations show that lysines have a much greater number of accessible rotamers than arginines of similar degree of burial in folded states of proteins. This finding suggests that lysines would preferentially entropically stabilize the native state. Indeed, we show in computational experiments that arginine-to-lysine amino acid substitutions result in noticeable stabilization of proteins. We then hypothesize that if evolution uses this physical mechanism as a complement to electrostatic stabilization in its strategies of thermophilic adaptation, then hyperthermostable organisms would have much greater content of lysines in their proteomes than comparably sized and similarly charged arginines. Consistent with that, high-throughput comparative analysis of complete proteomes shows extremely strong bias toward arginine-to-lysine replacement in hyperthermophilic organisms and overall much greater content of lysines than arginines in hyperthermophiles. This finding cannot be explained by genomic GC compositional biases or by the universal trend of amino acid gain and loss in protein evolution. We discovered here a novel entropic mechanism of protein thermostability due to residual dynamics of rotamer isomerization in native state and demonstrated its immediate proteomic implications. Our study provides an example of how analysis of a fundamental physical mechanism of thermostability helps to resolve a puzzle in comparative genomics as to why amino acid compositions of hyperthermophilic proteomes are significantly biased toward lysines but not similarly charged arginines.

## Synopsis

Comparative genomics sends us profound signals that are not easy to understand. For example, it is well known that proteins from hyperthermophiles are enriched with charged residues, but it has been a mystery why enrichment in positively charged amino acids is almost entirely due to lysines at the expense of very similar arginines. Here, the authors show that lysines (in contrast to arginines) exhibit significant residual dynamics in folded states of proteins, making the entropic cost to fold lysine-rich proteins less unfavorable compared with arginine-rich ones. Therefore, replacements of arginines by lysines provide additional thermal stabilization of proteins via entropic mechanism, making them positively charged residues of choice for evolutionary optimization of hyperthermostable proteins. Apparently, natural selection uses diverse physical mechanisms of thermal stability to achieve adaptation. This study provides an example of how better understanding of protein physics can help in solving genomic mysteries.

## Introduction

Enhancing the stability of globular proteins remains an important task of protein engineering and design [1,2]. The major mechanisms for increasing stability discovered so far vary from introduction of additional chemical bonds (e.g., disulfide bridges) or ion pairs [3–6] to increasing either the enthalpic free energy contributions by the optimizing of hydrophobic core interactions [7–11] or the entropic contributions by varying main-chain degrees of freedom in the unfolded state [12]. This repertoire of mechanisms relies on a variety of underlying physical principles for increasing protein stability [13]. The diversity of extreme environments and the long evolutionary history of organismal proteomes of extremophiles [14,15] suggest, in turn, many possible mechanisms of protein stabilization in response to the demands of the environment. Furthermore, the fact that each proteome contains a variety of structures and functions suggests that nature used all, even seemingly negligible, opportunities and their combinations for structure stabilization when adapting to extreme environmental conditions [15]. Here, we show how side-chain entropy in the native state can provide a mechanism of thermostabilization that is complementary to one of the major mechanisms of thermophilic adaptation, electrostatic interactions [3,4]. The analysis of statistics of rotameric states, together with computational mutation experiments, followed by high-throughput analysis of complete proteomes, reveals a previously unknown mechanism of stabilization via replacement of arginine residues with lysines. This substitution stabilizes the folded state, yet it preserves the charged nature of the substitution position, which may be important for other, perhaps functional, reasons. Thus, possible evolutionary advantages of this mechanism are as follows: (i) avoidance of sterically unfavorable contacts upon substitution, (ii) conservation of the similar-to-the-original (in terms of geometry and size) side-chains, and (iii) preservation of the positive charge and, as a consequence, important electrostatic interactions in the globule [3,4]. These subtle advantages exemplify the elegant work of natural selection and hint at the existence of other, yet undiscovered, mechanisms of protein adaptation.

## Results

### Monte Carlo Unfolding Simulations of Hydrolases H from *Escherichia coli* and *Thermus thermophilus*

The Gō model of protein folding is an idealized model in which the favorable interaction contact terms are exactly those found in the native structure [16]. In this model, the physico-chemical details of protein interactions are replaced by a generic contact energy term that is the same for all contacts between atoms that are found in contact in the native structure, though the complexity of the folded backbone and the side-chain conformations are preserved. It has been argued that such a model is a good representation of such aspects of protein energetics and folding, where non-native contacts do not play a massive role [16,17]. It remains unclear whether such an idealized model can quantitatively predict absolute folding transition temperatures. However, our results suggest that the Gō model predicts the transition temperature accurately enough to discriminate between proteins of thermophilic and mesophilic origin.

**Figure 1:**
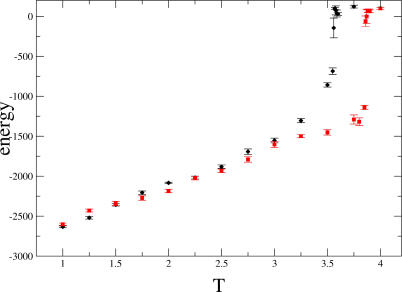
The Temperature Dependence of the Energy of Unfolding for Hydrolases, from *E. coli* (black rhombuses) and *T. thermophilus* (red squares) Every simulation of unfolding started from the native structure and lasted for 2 × 10^6^ MC steps; absolute temperature increment is 0.2 and 0.1 in the vicinity of transition temperature. The error bars represent mean square fluctuations of energy at each temperature calculated within productive part of a run when trajectory reached equilibrium after temperature increment.

Figure 1 shows the unfolding curves for the pair of meso/thermophilic hydrolase H from *Escherichia coli* and *Thermus thermophilus.* The Gō model correctly predicts a slightly higher transition temperature for the protein from thermophilic *T. thermophilus* compared with the one from *E. coli* (Figure 1)*.* Remarkably, the two unfolding curves coincide up to the transition, at which point they separate and then recombine at higher temperatures (Figure 1). Because the native states are enthalpically identical, and the folds are essentially the same, we surmise the origin of the transition temperature difference to be purely entropic. Specifically, given the nature of the Gō model, the entropic differences must arise from the different number of accessible rotamer states in different proteins. Calculation of average number of rotamers per residue in fully unfolded state [18] gives values of 12.0 and 11.4 for the mesophilic and the thermophilic proteins, respectively. These numbers thus demonstrate that the higher side-chain entropy in the unfolded state of mesophilic hydrolase is partially responsible for the fact that it unfolds at a lower temperature than the thermophilic structure.

### Lysine and Arginine: Archetypal Signal of Rotamer Entropy in Protein Stability

A careful look at the number of accessible states for each residue type in the folded state of hydrolases (Table 1) leads us to another interesting observation: although arginine and lysine are chemically similar and have the same *maximal* number of possible rotameric states, 81, they differ greatly in their rotameric accessibilities in the folded state.

**Table 1:**
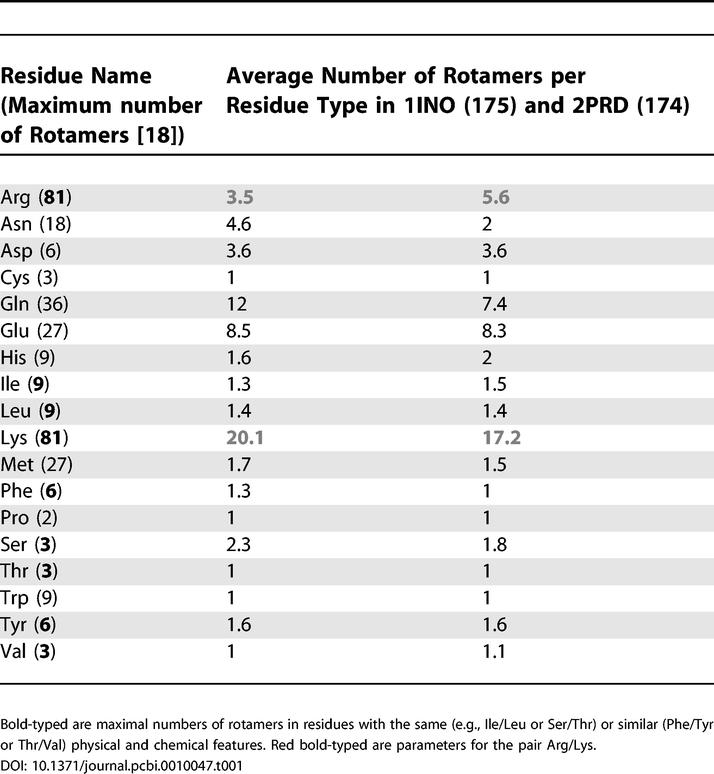
Average Number of Rotamers per Residue Type in the Folded State (Absolute Temperature T = 1) in Hydrolases H from *E. coli* and *T. thermophilus*

**Figure 2:**
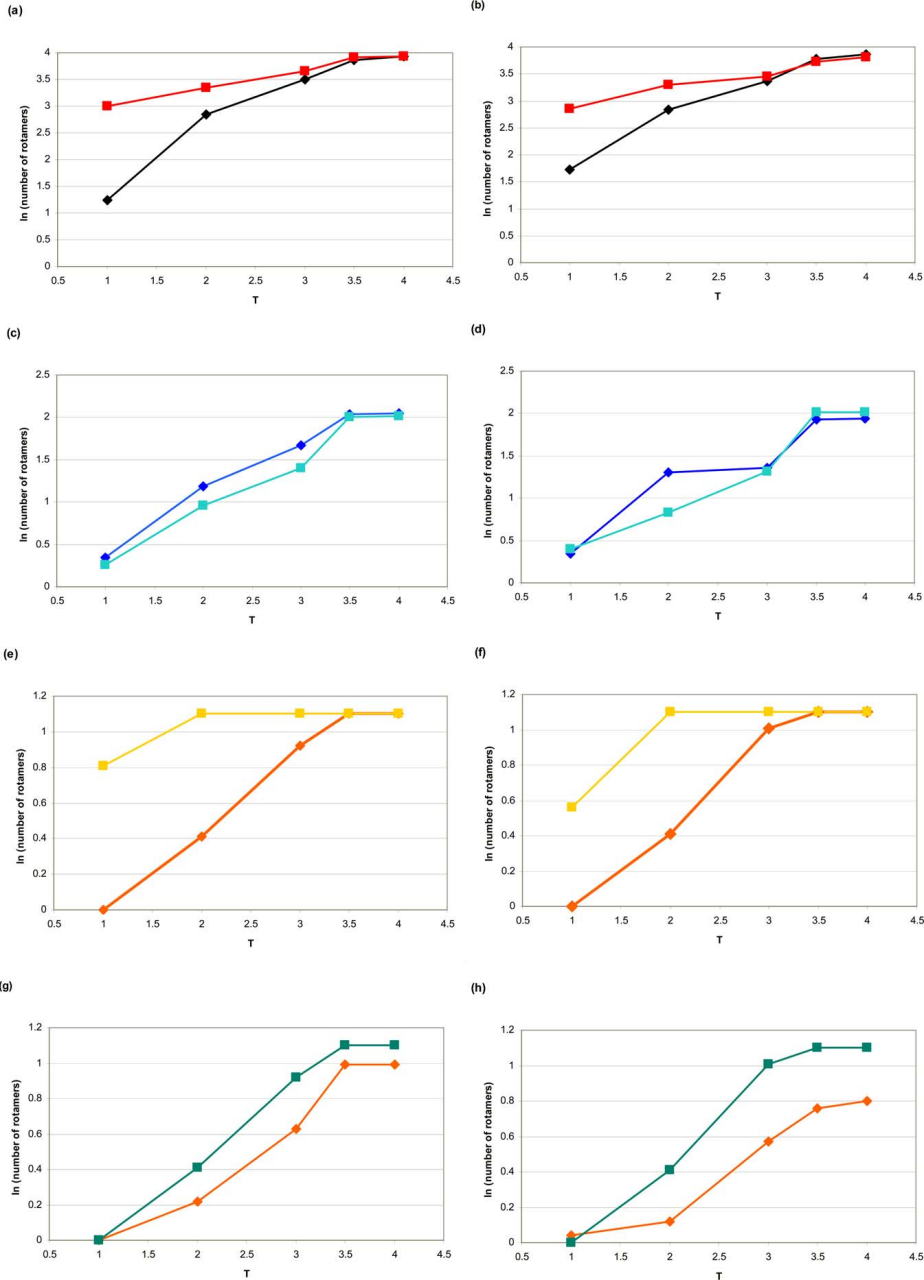
The Temperature Dependence of the Natural Logarithm of Number of Rotamers (A) Arg (black rhombuses) versus lysine (red squares) rotamers of hydrolase H from *E. coli*; (B) Arg (black rhombuses) versus lysine (red squares) rotamers of hydrolase H from *T. thermophilus*; (C) Leu (dark blue rhombuses) versus Ile (light blue squares) rotamers of hydrolase H from *E. coli*; (C) Leu (dark blue rhombuses) versus Ile (light blue squares) rotamers of hydrolase H from *T. thermophilus*; (E) Thr (orange rhombuses) versus Ser (yellow squares) rotamers of hydrolase H from *E. coli*; (F) Thr (orange rhombuses) versus Ser (yellow squares) rotamers of hydrolase H from *T. thermophilus*; (G) Thr (orange rhombuses) versus Val (green-blue squares) rotamers of hydrolase H from *E. coli*; (H) Thr (orange rhombuses) versus Val (green-blue squares) rotamers of hydrolase H from *T. thermophilus*.

There is a total of five groups of amino acid residues with the same maximal number of rotamers in unfolded state (Table 1): (i) Arg, Lys (maximal number of rotamers is 81); (ii) Glu, Met (27); (iii) Ile, Leu, His, Trp (9); (iv) Asp, Phe, Tyr (6); (v) Cys, Ser, Thr, Val (3). The amino acids lysine and arginine are both positively charged; both contain at least five heavy atoms in their side-chains. Both amino acids have four degrees of freedom in their rotatable bonds. The guanidinium group at the end of arginine displays resonance, and, as a consequence, has no internal rotational freedom. The salient difference between arginine and lysine is the fact that lysine is less bulky. Therefore, in the folded state, lysine may have slightly more freedom. Estimates of solvent accessibility of arginine and lysine do not reveal a substantial difference (unpublished data). As a control comparison, we use the pair isoleucine/leucine (each residue has a maximum of nine rotameric states and is similar to the other's physical and chemical properties). Using the Gō model for protein energetics, we sample the number of accessible rotamers as a function of temperature for the hydrolases from *E. coli* and *T. thermophilus* (Figure 2)*.* We approximate the entropy of the side-chain with the natural logarithm of the number of observed states in long equilibrium Monte-Carlo simulation. Figure 2 shows the temperature dependence of the natural logarithm of the number of rotamers for pairs Arg/Lys (Figure 2A and 2B), Leu/Ile (Figure 2C and 2D), Thr/Ser (Figure 2E and 2F), and Thr/Val (Figure 2G and 2H) in hydrolases H from *E. coli* and *T. thermophilis*. According to Table 1, lysine and arginine residues have different residual side-chain entropy. Lysine residues have many more rotamers in the folded state than arginine residues: on average, 20.1 and 17.2 versus 3.5 and 5.6 rotamers per residue of a particular type (Lys or Arg) in 1INO and 2PRD, respectively. The control group in this analysis is the pair Leu/Ile, which shows a highly similar temperature dependence of the number of rotamers (Figure 2C and 2D) for both proteins. Two last pairs, Thr/Ser and Thr/Val, confirm the role of the side-chain size and, as a consequence, its flexibility in providing number of accessible rotameric states. Each of Thr, Ser, and Val has a maximum of three possible rotamers and, thus, can be compared. Although both Thr and Ser are hydrophilic residues, Ser residues have a slightly greater number of rotameric states in the folded structure (at absolute temperature 1 in our temperature units used for MC simulations) as a result of its smaller side-chain. The hydrophilic/hydrophobic pair Thr/Val (Figure 2G and 2H) exhibit very similar behavior, stemming from the similarity of their side-chain geometries. This result is further substantiated by the temperature-dependence data for the pairs Val/Ser and Phe/Tyr. (The results for averaged temperature dependence, for residue types from both hydrolases, are presented in Figure S1). The bulky side-chains of both Phe and Tyr (Figure S1F) show practically the same temperature dependence, whereas in the pair Val/Ser (Figure S1E) the latter has slightly more rotamers in the folded state.

These results suggests that lysine and arginine provide an excellent platform to test a possible entropy-based mechanism of protein stabilization for both genomic and computational studies, for the following reasons: (1) they have similar physico-chemical properties, (2) they maintain the same physical and chemical features, and (3) they have similar rotamer entropies in the unfolded state but different rotamer entropies in the folded states (Table 1). Accordingly, we study the effects of side-chain entropy on protein stability for the chosen pair of types of amino acid residues, arginine and lysine [12,19–23].

### Statistics of Rotameric States in a Representative Set of Protein Structures

**Figure 3:**
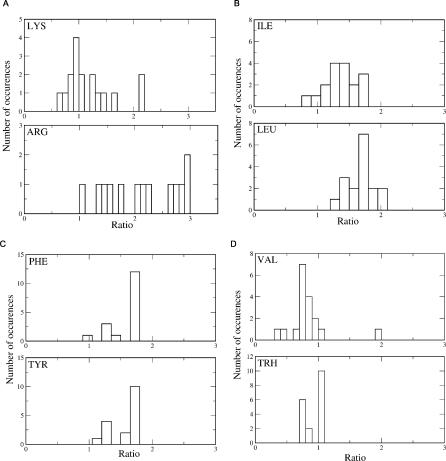
Distribution of the Ratios of the Number of Rotamers in Unfolded and Folded States in a Representative Set of Proteins Completely unfolded state is achieved at absolute temperature T = 4, folded state at T = 1. (A) Lys versus Arg; (B) Ile versus Leu; (C) Phe versus Tyr; (D) Val versus Thr. Upper histogram in each panel corresponds to T = 4, lower histogram corresponds to T = 1.

Let us consider a situation where residues with similar physical and chemical properties have a different number of rotamers in the folded state. The similarity of physical and chemical properties makes it possible to adjust stability due to entropic factor by mutating one residue type into another without changing the structure significantly. The first step to verify this mechanism is a statistical study of the difference in number of accessible rotamers for the folded and unfolded states. We analyzed the ratio of the number of rotamers (in natural logarithm units) at absolute temperature T = 4 (completely unfolded state) to that at T = 1 (folded state) for a representative set of 18 protein structures. Our results do not change from protein to protein and, as such, do not depend on the possible biases in the crystallographic quality of individual structures. Since we perform long runs of MC simulations (total of 10^7^ steps), which equilibrate our system and sample distinct rotamer states, we eliminate the memory of rotamer states in the original experimental structures. The difference between the number of Lys and Arg rotamers is also consistent for the representatives of different protein families, namely hydrolases, rubredoxins, ferredoxins, and chemotaxis protein (Table S1). Figure 3 shows histograms of ratios for the following pairs of amino acid residues: Arg/Lys (Figure 3A), Val/Thr (Figure 3B), and Phe/Tyr (Figure 3C).

Arginine and lysine show significantly different rotamer number ratios in the folded state distribution (Figure 3A; mean values of the distribution for lysine and arginine are 2.14 and 1.21 in natural logarithm units, respectively). Ratios for the pairs Leu/Ile, Val/Thr, and Phe/Tyr are very similar (Figure 3B–3D), with mean values of distributions 1.7/1.4, 0.85/0.97, and 1.62/1.58, respectively. These data corroborate that lysine residues contribute entropically to the change in equilibrium between the unfolded and folded states, whereas residues in pairs Leu/Ile, Val/Thr, and Phe/Tyr have similar number of rotamers in the folded state. As a next step, we prove the stabilizing role of lysine versus arginine in a direct computer simulation experiment.

### R/K Replacement Computational Experiment: Detecting Changes in Stability by Monte Carlo Unfolding Simulations

As stated above, both a statistical analysis of rotameric states and a comparative high-throughput analysis of complete proteomes demonstrate the particular role of lysine rotamers in protein thermostability. To demonstrate the stabilizing role of lysine residues, we make a replacement of type Arg/Lys and analyze the unfolding simulations in order to detect an anticipated increase in structure stability.

We replaced arginine residues with lysine residues in corresponding positions and locally minimized the resulting structures. We left the rest of the structure intact (see Materials and Methods) in order to influence the native structure as little as possible. The same local minimization was applied to the native structure.

Results of the replacements of arginine residues with lysine residues in both hydrolases H are as follows: in hydrolase H from *E. coli* position 43 (20 Arg-residue rotamers/31 Lys-residue rotamers in the folded state), 86 (2/5), 88 (4/12), and 171 (3/18); in hydrolase H from *T. thermophilus* position 24 (1/5), 43 (4/12), 114 (15/47), 156 (14/24), 158 (5/12), 166 (21/25), and 171 (3/10). We also analyzed combinations of Arg-to-Lys replacements in different positions in the structure. We found an increase of transition temperature in the replacement R171K and in combination of all R/K substitutions in positions 43, 86, 88, and 171 in mesophilic hydrolase from *E. coli*.

**Figure 4:**
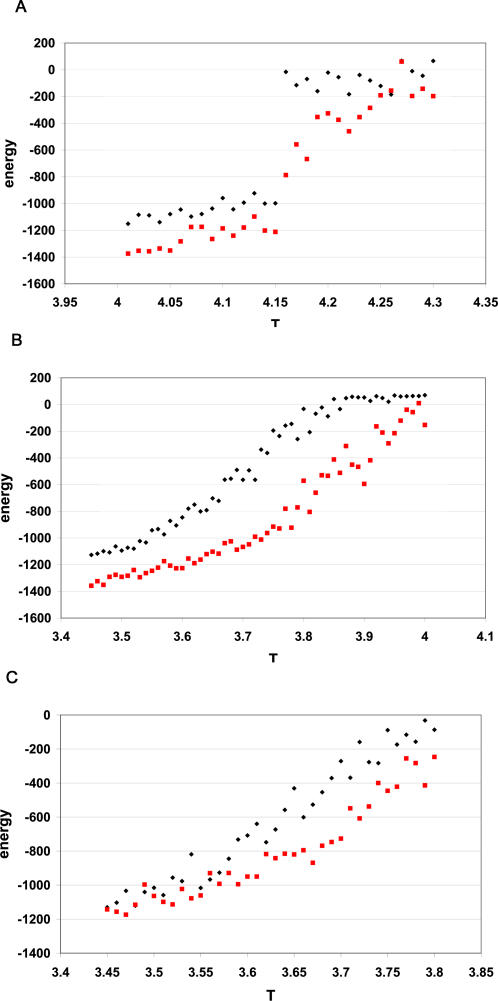
The Temperature Dependence of the Energy of Unfolding for Mutated (Red Squares) versus Original Hydrolases H (A) R171K mutant and wild-type of hydrolase H from *T. thermophilus*; (B) R171K mutant and wild-type of hydrolase H from *E. coli*; (C) R43,86,88,171K mutant and wild-type of hydrolase H from *E. coli*.

Figure 4A and 4B shows a plot of the temperature dependence of the energy in unfolding simulations of structures with replacement R171K in hydrolases H from *E. coli* and *T. thermophilus* [24–26]. Though there is a slight increase in the enthalpic term in the modified (R171K) structure of thermophilic hydrolase (3,321 native contacts in the modified structure versus 3,246 in the original, according to the Gō model), and an increase in the number of rotamers in the modified structure, there is no indication of a change in the transition temperature in unfolding simulations (Figure 4A). Similar replacements in the structure of mesophilic hydrolase H from *E. coli,* on the other hand, cause a change in the transition temperature of approximately 0.1 in absolute units (2.6%). The increase in the number of native contacts in the modified structure (3,226 in modified versus 3,131 in original) accounts for 3% of the difference in transition temperature; entropic factors do not play a stabilizing role in this case (5.38 and 5.34 rotameric states per residue in original unmutated structures, respectively). We detected an increase in the stability of the structure when all arginine residues (positions 43, 86, 88, and 171) were replaced by lysine residues. Taking into account both the decrease in the enthalpic term in the modified structure (3,083 native contacts versus 3,102 in the original, or approximately 0.6% loss) and the simultaneous increase in the transition temperature by 0.05 of absolute units (gain of 1.3%) gives a total increase of 2% in stability, which we conclude to be an effect of entropy stabilization of the structure. The number of rotamers per residue increases from 5.41 in the original to 5.62 in the mutated structure, a 4% difference, which, taking into account the roughness of the estimate, corroborates an increase in stability. The absence of a stabilizing effect of replacements in thermophilic hydrolase H from *T. thermophilus* can be explained by the high stability of the original protein, as demonstrated earlier [24–26].

**Figure 5:**
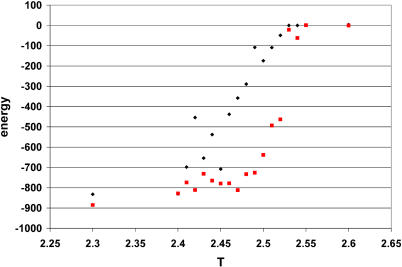
The Temperature Dependence of the Energy of Unfolding for R24,26,53,58,74,80,87,95K Mutant Compared with the Original Structure of Cytochrome C from *R. sphaeroides*

We performed a similar experiment with a smaller protein to improve sampling. Cytochrome C from *Rhodobacter sphaeroides* [27] contains 112 amino acid residues, with positions 24, 26, 53, 58, 74, 80, 87, and 95 occupied by arginine residues. Simulations reveal the following variations in the number of rotamers in each position upon replacement with Lys: position 24 (8/16), 26 (15/17), 53 (37/38), 58 (5/22), 74 (2/6), 80 (43/43), and 95 (8/52). Replacement in individual positions did not reveal an increase in stability. However, simultaneous substitution of all arginine residues by lysines led to a noticeable increase in transition temperature, while the enthalpic term decreased by 0.5% (1,607 native contacts in the modified structure, compared with 1,615 in the original). Figure 5 shows the temperature dependence of the energies, averaged over five runs (each 5 × 10^7^ MC steps). The difference between the transition temperature of the original and the modified structures is ΔT = 0.07 absolute units (3%) increase, which translates into a 3.5% increase in stability when the unfavorable change in enthalpy is taken into account. Indeed, the mutated structure demonstrates an increase in the entropy of the folded state, 5.03 versus 4.56 rotameric states per residue in the original structure.

These data show that lysine residues contribute greatly to the stabilization of folded states of proteins, compared with their peer positively charged arginine, whereas residues in pairs Leu/Ile, Val/Thr, and Phe/Tyr have similar number of rotamers in the folded state. It is possible that this mechanism of stabilization is employed by nature in its strategies of thermophilic adaptation. If this is the case, it should be manifest in comparative genomics analysis in greater content of lysines in hyperthermophiles compared with mesophiles and, importantly in bias toward Arg-to-Lys substitutions from mesophiles to hyperthermophiles.

### Analysis of Complete Proteomes

#### Amino acid composition biases in hyperthermophilic proteomes.

We performed quantitative analysis on 38 mesophilic and 12 hyperthermophilic proteomes. (For a list of the genomes used, see Tables S2 and S3.)

**Figure 6:**
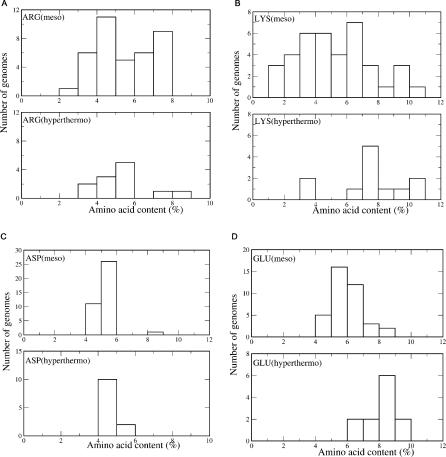
Histograms of the Content of Charged Amino Acid Residues in Hyperthermophilic Genomes Compared with Mesophilic Genomes Top histogram shows percentage of each residue in mesophilic genomes; bottom histogram, in hyperthermophilic genomes. A total of 12 hyperthermophilic and 38 mesophilic genomes were analyzed (for the complete list, see Tables S1 and S2). (A) Arg; (B) Lys; (C) Asp; (D) Glu.

It has been demonstrated earlier that mesophilic proteins posses rather limited stability [28]. In the case of (hyper)thermophilic proteins, stabilization should be much stronger and, thus, it requires concerted contribution from many possible mechanisms. For this reason, we intentionally considered only hyperthermophilic proteomes in order to capture the most pronounced sequence biases associated with the extreme thermal stability of hyperthermophilic species. Figure 6 and Figure S2 show sets of composition histograms for two types of residues charged and hydrophilic, respectively, presumably associated with variations in thermal stability. While in thermophilic species the percentage of polar residues is high [29], this percentage is the same or even smaller in hyperthermophilic organisms (for instance, Glu, Ser, Thr; see Figure S2). In the case of charged residues, we observe clear under-representation of Asp and His and an increase of Glu (Figure 6) in hyperthermophilic organisms. Increase of the Glu content is usually explained by its longer side-chain, which provides more opportunities for ion interaction [30,31]. It should be noted that increase of Glu at the expense of Asp can be a consequence of higher entropic contribution from Glu compared with Asp. However, Glu also has a longer side-chain, which may naturally increase its enthalpic contribution. Thus, the role of the toward Glu deserves separate consideration with careful analysis of both enthalpic and entropic effects. In addition to the earlier-detected increase of total content in the Arg/Lys pair [30], we found that in ten of the 12 hyperthermophilic genomes lysine content is much higher (not less than 6%), whereas arginine content is distributed evenly mostly between 3% and 6% (Figure 6A and 6B). The dominance of arginine in the pair Arg/Lys in proteomes of *Methanopyrus kandleri* and *Aeropyrum pernix* is an exception due to the high GC content in these genomes [32,33]. Mean values for the percentage of Arg, Lys, His, Asp, and Glu in mesophilic and hyperthermophilic organisms (excluding *M. kandleri and A. pernix*), along with *p*-values according to binomial distribution calculated for the pair of archetypal representatives of each group, *E. coli* and *Pyrococcus furiosus* (Table S4) are: Arg, mesophilic/hyperthermophilic genomes, 5.46/4.94 (*p* = 8 × 10^−11^); Lys, 5.35/8.48 (*p* < 10^−14^); Asp, 5.28/4.72 (*p* < 10^−14^); Glu, 6.1/8.42 (*p* < 10^−14^). Thus, ten of the 12 hyperthermophilic organisms show difference in the preference for charged residues in mesophilic and hyperthermophilic genomes. (Note that for Arg and Asp, the difference is inverse: there are *more* such groups in mesophiles than in thermophiles.) In particular, we detected an increase of lysine content at the expense of arginine content.

#### Comparative analysis of hyperthermophilic versus mesophilic proteomes.

A persistently high percentage of Arg+Lys, though biased in most of the proteomes toward increased lysine content, along with the similarity in physical and chemical features of these residues suggests an examination of substitutions of types R/K versus K/R in the alignment of mesophilic sequences (here, *E. coli*) versus hyperthermophilic ones. We started from the following hypothesis: if, as stated elsewhere [30], only the total content of arginine plus lysine residues matters in determining the stability of hyperthermostable proteins, then there should be no preference for one of the residues (Lys) over the other one (Arg). We used sequences of five hyperthermophilic archaea *(Aeropyrum pernix, Methanococcus jannaschii, Nanoarchaeum equitans, P. furiosis,* and *Sulfolobus tokodaii)* and one hyperthermophilic bacteria *(Aquifex aeolicus).* Outputs of BLAST alignments were used for comparison of sequence substitutions that favor one or the other residue in each pair (Table 2). Our data are presented in Table 2. The number before the slash is the percentage of amino acid residues in the mesophilic sequence, e.g., Leu that was replaced by the other amino acid in the hyperthermophilic sequence, e.g., Ile. The number after the slash reflects the same data for the opposite replacement, e.g., Ile, in the mesophilic sequence by Leu in the hyperthermophilic sequence. The control groups here are the pairs Leu/Ile and Ser/Thr; both residues in each pair are hydrophobic or polar, and both have the same maximal number of possible rotamers, nine and three, respectively. In all alignments of *E. coli* sequences against those from one of the hyperthermophilic genomes, we obtained equal or very similar numbers of residues substitutions (numbers in parenthesis show ratio of forward to back substitutions). The exceptions are pairs LI/IL and RK/KR in *A. pernix,* which show bias in the opposite direction explained by the GC content. Unlike the above control groups, the pairs RK/KR demonstrate a remarkable bias toward replacement of arginine in the mesophilic sequence with lysine in the hyperthermophilic sequence (at least 1.6 times in *P. furiosis,* and up to almost four times in *N. equitans*). *p*-Values (calculated according to χ^2^ criteria) show a statistically significant preference for the arginine-to-lysine substitution as opposed to the reverse one. This challenges the idea that arginine and lysine play the same role in thermostability [30]. Therefore, our comparative genomics analysis strongly supports the conclusion that lysine has a particular or even exceptional role in protein stabilization [31].

**Table 2:**
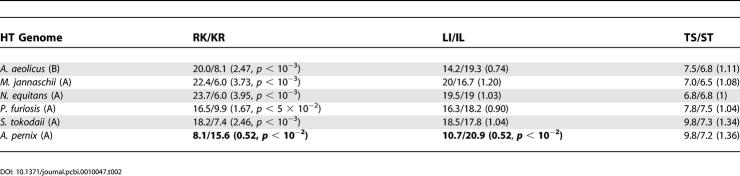
Percentage of the Forward/Back Replacements in Alignments of Hyperthermophilic Genomes against Mesophilic One (*E. coli*)

Recently, Jordan et al. [34] attempted to find “universal trend of amino acid gain and loss in protein evolution.” They found two major groups of amino acids: Cys, Met, His, Ser, and Phe; and Pro, Ala, Glu, and Gly, that are accrued and consistently lost. Their major conclusion is that, in agreement with earlier developed amino acid chronology [35], “all amino acids with declining frequencies are thought to be among the first incorporated into genetic code; conversely, all amino acids with increasing frequencies, except Ser, were probably recruited late.” Importantly, amino acid chronology proposed in [34] reflects early stages of protein evolution [35,36], and, technically, it was developed on the basis of codon chronology. The latter started from the GC-rich codons, as first codons are believed to be more thermostable than all repertoire of codons, and corresponding amino acids emerged as result of wobble and transition mutations. Thus, both amino acid chronology and universal trend of amino acid gain and loss in protein evolution demonstrate generic connection between DNA and proteins composition and its evolution. Our findings, however, are of a different nature. Contrary to “universal trend,” which does not discriminate between meso- and (hyper)thermophilic organisms, bias toward Lys residues is a statistically significant trend of hyperthermophilic proteomes (Figure 6). Thus, we discovered here a new mechanism of thermophilic adaptation that happens on the level of amino acid composition and originates from the specific physical chemical features of arginine/lysine residues. Finally, entropic mechanism of stabilization is complementary to generic amino acid chronology, and it demonstrates work of natural selection in order to reach adaptation to extreme environmental condition.

## Discussion

### Thermodynamical Models of Protein Stability and the Enthalpy/Entropy Relationship as a Manifestation of a Variety of Stabilizing Factors

Most of the data on structure thermostability and its major factors come from experiments aimed at analyzing the role of individual contributors, such as hydrophobic, van der Waals, electrostatic [3,4], and other physical forces [13,14]. This determines a common computational approach to the analysis of protein thermostability: a limited dynamic or static model with a detailed Hamiltonian that partitions the forces into distinct classes [21,37,38].

The approach we presented here straddles the way between a complete description of folding and the limited dynamic models presented in previous studies. We employ a Gō model [16,17], which enables us to account for the enthalpically relevant terms, albeit in a coarse-grained manner. The Gō model also permits us to account accurately for the various entropic contributors to the folding free energy, namely the backbone entropy and side-chain entropies. Finally, and most importantly, the simplicity of the model means that we are able to probe these various free energy effects with multiple folding runs relatively easily. In short, this approach makes it possible to examine the generic aspects of thermodynamics of thermostability.

Our results show the utility of Monte Carlo unfolding simulations with the Gō model as a way to detect the relative contributions of the free energy components, enthalpy and entropy. Furthermore, the description of the unfolding simulation in terms of the enthalpy/entropy relationship highlights the differences in the contribution of different types of amino acid residues to the entropic part of the free energy balance of a protein (Table 1). We found a difference in the number of accessible rotamers *in folded state* despite the fact that these residues were naively expected to be fully fixed in native states, i.e., all have only one rotamer available in the native state. Logarithm of the ratio of the number of rotamers in the folded and unfolded states gives us the entropy difference upon folding for each residue (Figures 2 and 3). These data demonstrate significantly higher entropy of lysine residues in folded states compared with those of arginine.

We demonstrate here that our top-down approach, from analysis of thermodynamic quantities to discovery of concrete physical processes that give rise to the observed thermodynamic phenomena, can not only detect differences in the free energies of stabilization, but also reveal novel mechanisms of stabilization via the rotamer entropic effect.

### Genomic Motivation for the Novel Mechanism of Thermostability

To validate a model of protein stability on the genomic and proteomic level, it is important to find particular expected compositional and sequence biases by means of massive high-throughput analysis. Even if the bulk of the protein in the organism exhibits a particular mechanism of stabilization according to the mechanism of adaptation commonly developed in the proteome [39–41], one or a few proteins may rely on a different/additional mechanism developed under specific environmental conditions. What additional information can we glean from the proteome analysis? First, amino acid compositional analysis reveals a bias toward lysine residues in the pair Arg/Lys, typical for the genomes of hyperthermophiles. Such analysis also a bias toward Lys and Glu in hyperthermophilic proteomes, whereas Asp and His are unfavorable in these organisms. The only exceptions are two hyperthermophilic genomes, *A. pernix* and *M. kandlerii,* whose preference for arginine residues is a direct consequence high GC content [31,33,42]. Second, comparative analysis of hyperthermophilic and mesophilic (here, *E. coli*) proteomes reveals an enrichment of lysine content at the expense of the arginine.

Though bias in amino acid composition toward increasing charged residues is well documented in earlier works [29–31,43–46], the difference in the frequencies of arginine and lysine residues has not been explained unequivocally [30,47].

There is a strong belief that GC content is the major factor in ensuring survival and selective advantages for extremophiles, in particular thermophiles, due to high thermostability of GC pairs [46]. Assuming that this explanation is correct, one would expect (hyper)thermophiles to select arginine over lysine. Arginine is encoded by six codons, four of which (CGU, CGC, CGA, and CGG) are GC-rich, whereas lysine is encoded by two codons (AAA and AAG). Moreover, arginine has a higher charge, which means it forms better salt bridges [47]. Surprisingly, this expectation is confirmed in only a very few cases, for instance in *A. pernix* and *M. kandlerii*; whereas in the majority of other hyperthermophilic organisms, we observe significant increase in lysine content, which typically anticorrelates with GC content. Furthermore, as we demonstrated here, lysine content partially increases due to direct replacement of arginine residues (Table 2), which points out the obvious advantage that lysine residues have over arginine. One could argue that (i) composition effect alone can account for the higher substitution rate of Arg/Lys, or that (ii) there was a particular common ancestor enriched by Lys, and the specific compositional bias in contemporary proteins that we observe is due to phylogeny. But the unfolding simulations, the statistical data on rotameric states, and the genomic evidence all point to the advantage of lysine over arginine when thermostability is important. Furthermore, we see excess of lysine only in hyperthermophilic organisms, regardless of their loci on the phylogenetic tree (e.g., archaea and bacteria). Lysine still has some entropic freedom, even in the folded state of a protein, due to its smaller size. In comparison, arginine, with its bulky guanidinium group, does not have the same freedom, and its possible enthalpic advantage is compensated by the drawback of packing of two closely located charges [48].

### Adaptation to High Temperatures as a Complex Effect of Different Types of Interactions

We discovered here a novel mechanism of structure thermostabilization that relies on side-chain rotamer entropy [19,23]. To single out the potential effects of rotamer entropy, we compared pairs of amino acid residues with similar physical and chemical properties and the same maximal number of rotameric states. The difference in the rotamer entropy of each pair of residues must, then, be a result only the difference in the rotameric entropy of their side-chains. Statistical data of accessible rotameric states (see Figures 2 and 3 and Table 1) show substantial entropy for lysine residues in both folded and unfolded states, whereas arginine has a significantly decreased side-chain freedom in the folded state. Preference for the lysine is also supported by the genomic data (see Figure 6 and Table 2) and illustrated by the computational mutation experiment (Figures 4 and 5).

In general, just a few mutations can make the difference between a mesophilic protein and its (hyper)thermophilic counterpart. Stability is reached by fine-tuning sequences and structures, rather than by drastic rearrangement. Moreover, in the case of hyperthermophilic proteins, practically all possible means of stabilization appear to be utilized. Any additional element of stabilization must both preserve the already-achieved level of stability and provide additional stabilization by invoking only minor modifications in sequence and structure. Arg/Lys replacement satisfies both of these conditions and, thus, exemplifies using the entropic contribution while simultaneously preserving the charged nature of the residues, which is important for other mechanisms of stabilization, such as the electrostatic [49,50]. Indeed, as it has been thoroughly demonstrated elsewhere [3,4], electrostatics is one of the major factors of thermostability. The entropic mechanism discovered in this work serves as an important complementary factor that provides additional stabilization when the repertoire of other mechanisms has already been possibly exhausted [5,6].

The novel mechanism of thermal stabilization reported here is unique in that it relies not only on the physical and chemical properties of a residue, but also on its dynamics in folded state affecting its entropic contribution. And because the effect is small, it can be revealed only in careful simulations and genomic comparisons. The important pedagogical point we draw from this result is that the study of protein stability on individual proteins using current state-of-the-art energy functions may result in missing subtle thermodynamic evolutionary signals that only become apparent in high-throughput analysis of proteomes and genomes.

## Materials and Methods

### Statistics of rotameric states.

The number of accessible rotamers in the folded (T = 1) and fully unfolded (T = 4, see Figure 1) states for a representative set of proteins was calculated. The temperature dependence for the number of accessible rotamers in hydrolases H from *E. coli* and *T. thermophilus* was calculated at absolute temperatures T = 1, 2, 3, 3.5, and 4. Structure coordinates were recorded at every 10^5^ MC steps for a total of 10^7^ steps. The number of rotamers for every residue were determined as an average over 100 snapshots.

We used the following PDB structures to collect statistics of rotameric states (see also Table S1): (1) hydrolases, (2) rubredoxins, (3) 2Fe-2S ferredoxin, (4) 4Fe-4S ferredoxin, and (5) chemotaxis protein.

Statistics of rotameric states in original and mutated structures of hydrolases and Cytochrome C were collected from recorded structures at every 10^4^ MC steps for a total of 10^7^ steps done for every original/mutated structure (1,000 snapshots). Our results do not depend on crystallographic quality of the structures, and we obtain consistent data for the following reasons: (i) we work with high-resolution structures; and (ii) most important, that we do long runs of MC simulations which equilibrate a system and, thus, eliminate any possible discrepancies in original structures.

### High-throughput sequence analysis.

We used the BLAST program [51] to create a set of pair-wise alignments with significant *e*-value (*e* = 0.05) using the substitution matrix BLOSUM62. We chose only sequences that had gaps of length 3 or less, and full alignment length of 45 residues or more.

### Molecular dynamic minimization.

We used the CHARMM program [52] to minimize the structure upon Arg/Lys replacement. CHARMM minimization was done using the following procedure. Hydrogen coordinates were calculated by bond geometry and inserted into the starting structure; SHAKE was turned on for updating hydrogen positions. A generalized-Born solvation energy function (GBorn) and a dielectric constant with linear distance-dependence were used for dynamics. The residue of interest, and all atoms within a 5-angstrom radius of any atom in that residue, were permitted to move with CHARMM degrees of freedom to ensure that the mutated residue could repack locally. The dynamics simulation was initially constrained to the native state using a harmonic potential. The artificial harmonic constraint was reduced to zero slowly over consecutive cycles of adopted-basis Newton-Raphson minimization. To detect the effects of mutation, we minimized both mutated and original structures with the same protocol in order to use the latter one as a control.

### Unfolding Monte Carlo simulations of modified proteins.

Unfolding simulations were performed using an all-atom Gō model developed earlier [53]. In the Gō interaction scheme, atoms that are neighbors in the native structure are assumed to have attractive interactions. Hence, the Gō model of interactions is structure-based. Every unfolding run consists of 2 × 10^6^ steps in the unfolding simulations of hydrolases (Figure 1) and their mutants (Figure 5A–C), and 5 × 10^7^ steps in the case of Cytochrome C (Figure 6). The move set is one backbone move followed by one side-chain move [53].

## Supporting Information

Figure S1 The Temperature Dependence of the Natural Logarithm of the Number of Rotamers Averaged over Respective Values in Hydrolases H from *E. coli* and *T. thermophilus*
(A) Arginine (black rhombuses) versus Lys (red squares) rotamers; (B) Leu (dark blue rhombuses) versus Ile (light blue squares); (C) Thr (orange rhombuses) versus Ser (yellow squares); (D) Thr (orange rhombuses) versus Val (green-blue squares); (E) Val (green-blue rhombuses) versus Ser (yellow squares); (F) Phe (green-blue rhombuses) versus Tyr (orange squares).
(14 KB PDF)

Figure S2 Histograms of the Content of Polar Amino Acid Residues in Hyperthermophilic Genomes Compared with Mesophilic Ones Top histogram shows percentage of respective residue in mesophilic genomes; bottom histogram, in hyperthermophilic ones. Total of 12 hyperthermophilic and 38 mesophilic genomes were analyzed (for the complete list, see Tables S1 and S2). (A) Asn; (B) Gln; (C) His; (D) Ser; (E) Thr; (F) Tyr.
(766 KB TIF)

Table S1 Set of Proteins Used in Collecting Comparative Rotamer Statistics (44 KB DOC)

Table S2 List of Mesophilic Genomes Total of 38 genomes. Columns are as follows: first, genome accession number in NCBI database of complete genomic sequences; second, name of the organism; third, Life Kingdom (A, archaea; B, bacteria); fourth, size of the proteome in number of protein coding sequences.
(57 KB DOC)

Table S3 List of Hyperthermophilic Genomes Total of 12 genomes. Columns are as in Table S2.
(33 KB DOC)

Table S4 Expected (on the Basis of the Occurrence in *E. coli,* Column 4) and Observed (Column 5) Frequencies of Charged Amino Acid Residues in *P. furiosis*
Diff in σ, difference in number of standard deviation between respective expected and observed values. The null model used to calculate *p*-values represents random uncorrelated distribution of charged amino acids over proteomes resulting in binomial distribution for the content of each type of amino acids, from which *p*-values were calculated.
(29 KB DOC)

### Accession Numbers

The Protein Data Bank (http://www.rcsb.org/pdb/) accession numbers for products used in this paper are 2Fe-2S ferredoxin (4FXC, 1FRR, 1FRD, 1DOI, and 2CJN); 4Fe-4S ferredoxin (1FCA, 1DUR, 1IQZ, and 1VJW); chemotaxis protein (3CHY, 2CHF, and 1TMY); Cytochrome C (1DW0); hydrolases (1INO [from *E. coli*] and 2PRD [from *T. thermophilus*]); rubredoxins (1RDG, 5RXN, 8RXN, and 1CAA).
