# Supplementary figures and images for: Entropic Stabilization of Proteins and Its Proteomic Consequences
Source: PLoS Comput Biol. 2005 Sep 30;1(4):e47. doi: 10.1371/journal.pcbi.0010047 (PMC1239905; doi:10.1371/journal.pcbi.0010047)

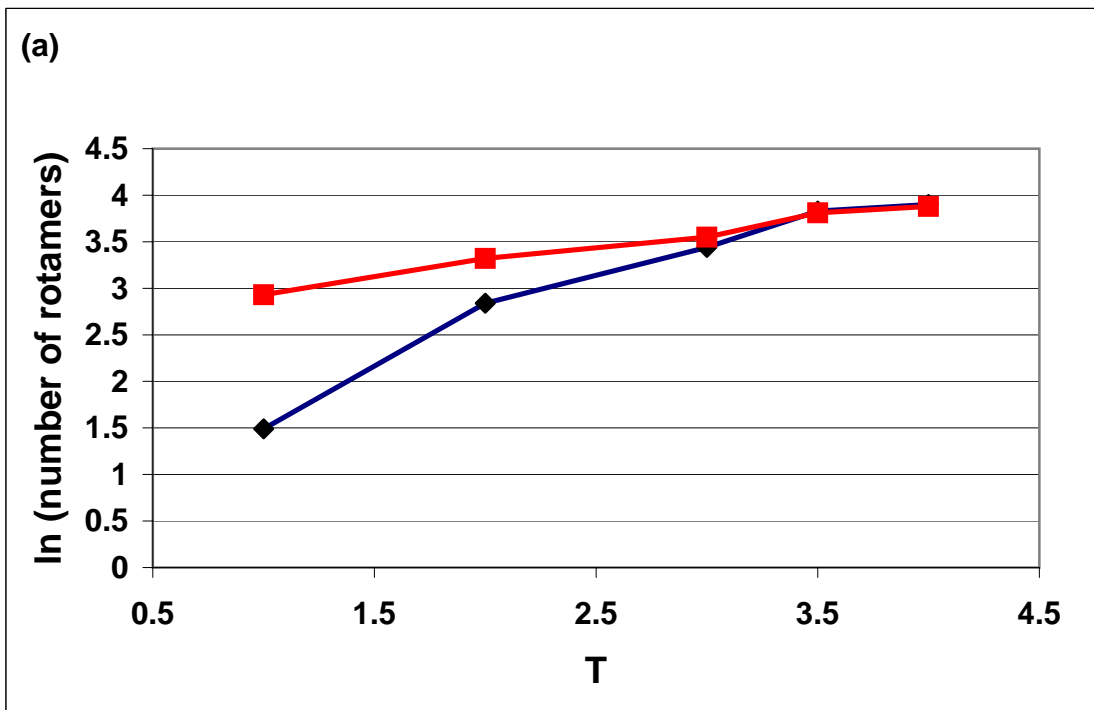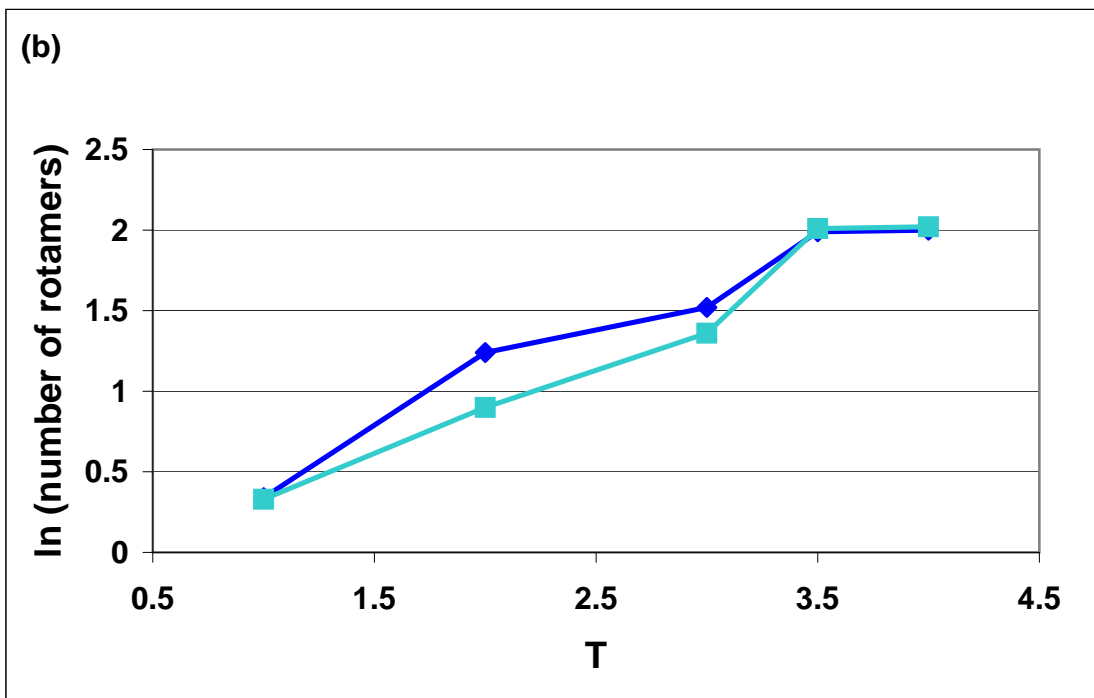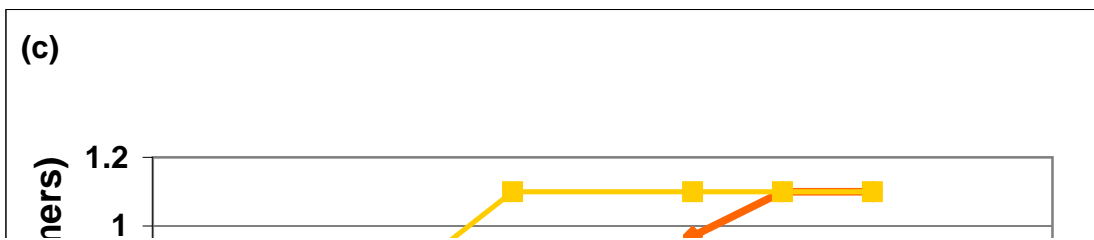

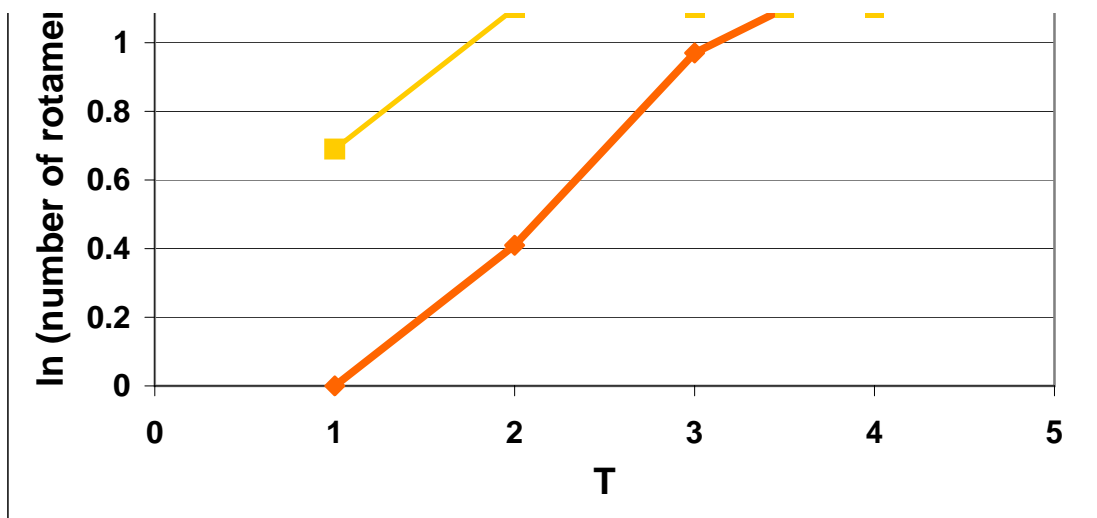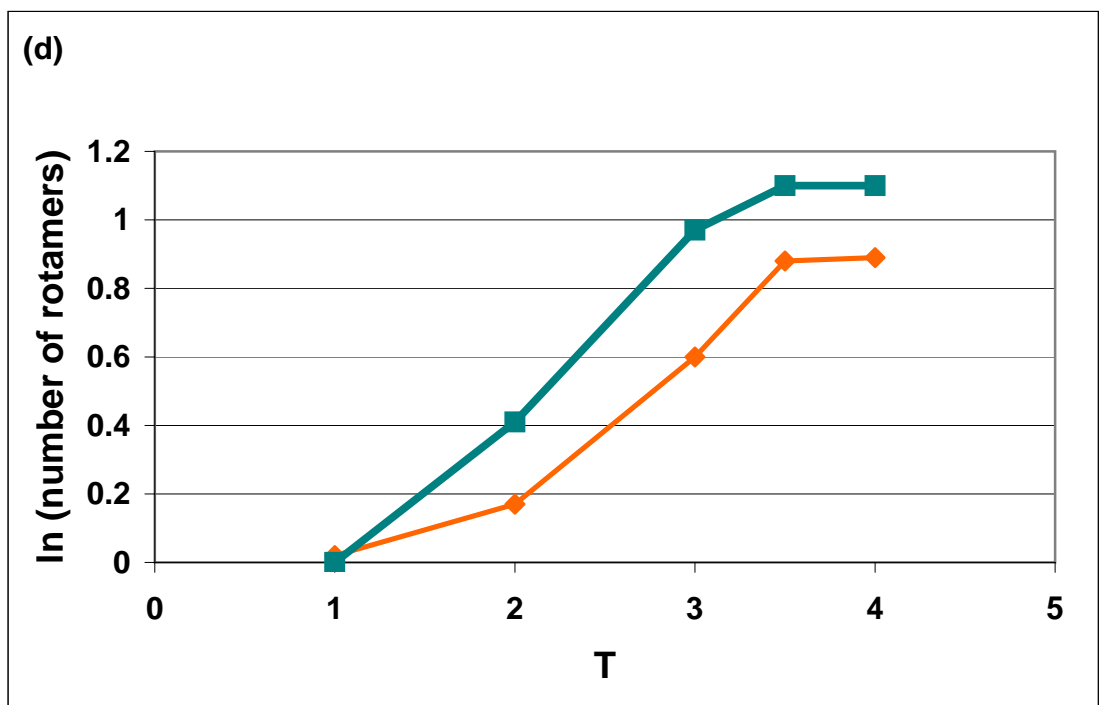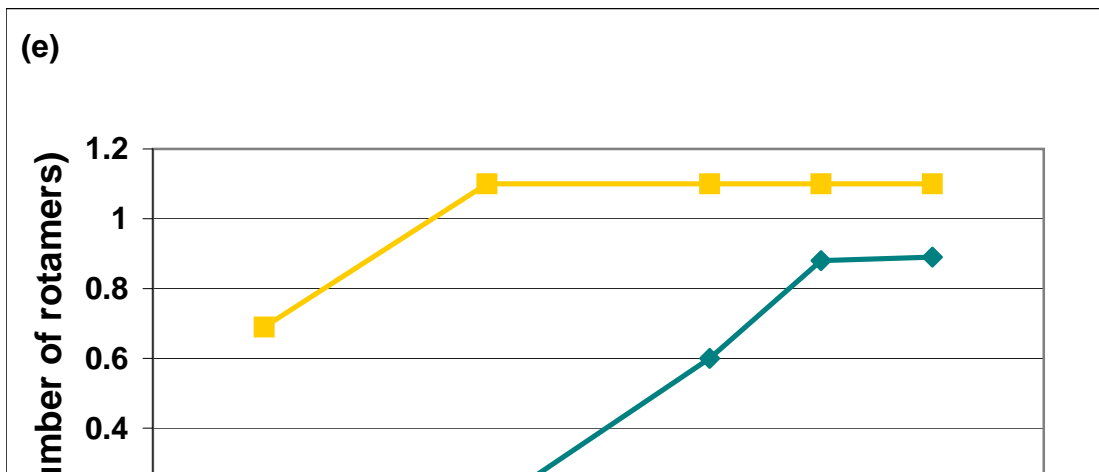

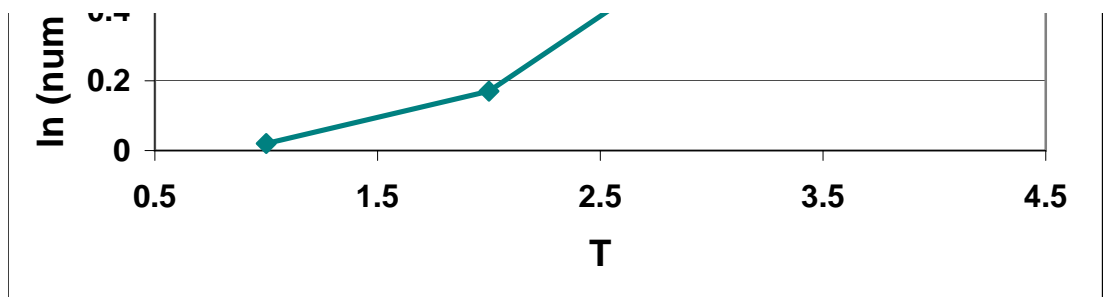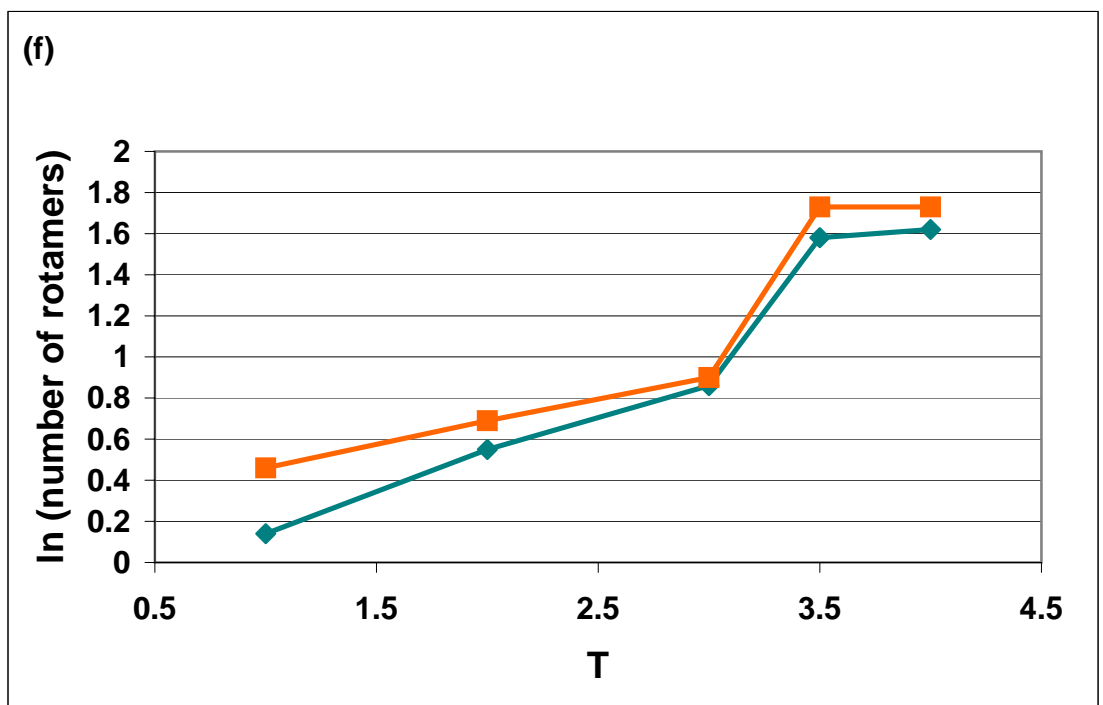

Supplement: Figure S1 — (A) Arginine (black rhombuses) versus Lys (red squares) rotamers; (B) Leu (dark blue rhombuses) versus Ile (light blue squares); (C) Thr (orange rhombuses) versus Ser (yellow squares); (D) Thr (orange rhombuses) versus Val (green-blue squares); (E) Val (green-blue rhombuses) versus Ser (yellow squares); (F) Phe (green-blue rhombuses) versus Tyr (orange squares). (14 KB PDF) [file pcbi.0010047.sg001.pdf]

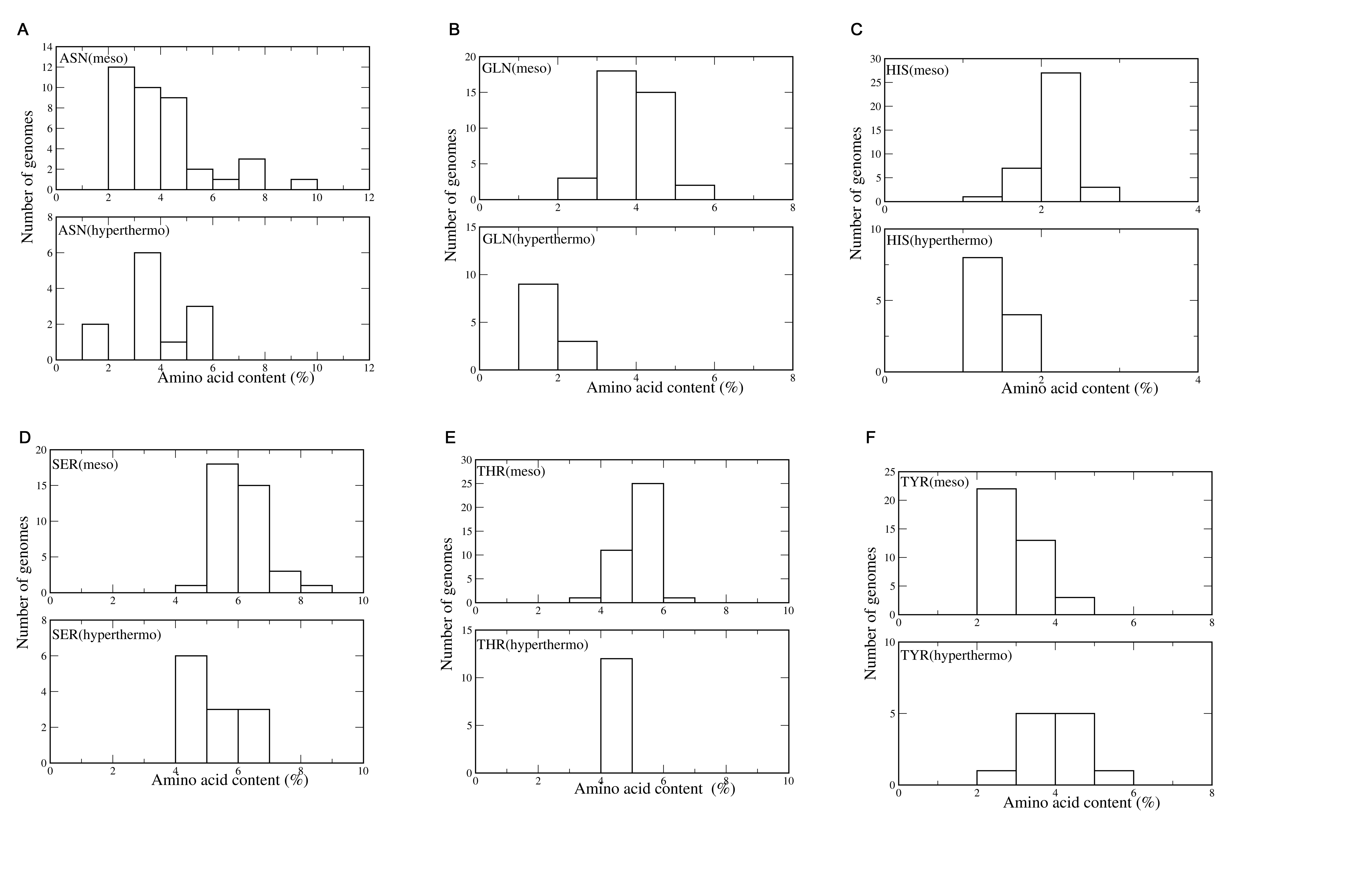

Supplement: Figure S2 — Top histogram shows percentage of respective residue in mesophilic genomes; bottom histogram, in hyperthermophilic ones. Total of 12 hyperthermophilic and 38 mesophilic genomes were analyzed (for the complete list, see Tables S1 and S2). (A) Asn; (B) Gln; (C) His; (D) Ser; (E) Thr; (F) Tyr. (766 KB TIF) [file pcbi.0010047.sg002.tif]
