# Supplementary material for: Entropic Stabilization of Proteins and Its Proteomic Consequences
Source: PLoS Comput Biol. 2005 Sep 30;1(4):e47. doi: 10.1371/journal.pcbi.0010047 (PMC1239905; doi:10.1371/journal.pcbi.0010047)
Supplement: Table S1 — (44 KB DOC) [file pcbi.0010047.st001.doc]

**Table S1**

| **Protein (number of residues)** | **Source** |
| --- | --- |
|  |  |
| **Hydrolase** |  |
| 1INO (175) | *E. coli* |
| 2PRD (174) | *T. thermophilus* |
|  |  |
| **Rubredoxin** |  |
| 1RDG(52) | *D. gigas* |
| 5RXN (54) | *C. pasteuranium* |
| 8RXN (55) | *D. vulgaris* |
| 1CAA (53) | *P. furiosus* |
|  |  |
| **Ferredoxin (2FE-2S)** |  |
| 4XFC (98) | *S. platensis* |
| 1FRR (95) | *E. arvense* |
| 1FRD (98) | *Anabaena PCC7120* |
| 1DOI (128) | *H. marismotui* |
| 2CJN (97) | *S. elongates* |
|  |  |
| **Ferredoxin (4FE-4S)** |  |
| 1FCA (55) | *C. acidiurici* |
| 1DUR (55) | *P. asaccharolyticus* |
| 1IQZ (81) | *B. thermoproteolyticus* |
| 1VJW (59) | *T. maritima* |
|  |  |
| **Chemotaxis protein** |  |
| 3CHY (128) | *E. coli* |
| 2CHF (128) | *S. typhimurium* |
| 1TMY (118) | *T. maritima* |
