# Supplementary material for: Entropic Stabilization of Proteins and Its Proteomic Consequences
Source: PLoS Comput Biol. 2005 Sep 30;1(4):e47. doi: 10.1371/journal.pcbi.0010047 (PMC1239905; doi:10.1371/journal.pcbi.0010047)
Supplement: Table S2 — Total of 38 genomes. Columns are as follows: first, genome accession number in NCBI database of complete genomic sequences; second, name of the organism; third, Life Kingdom (A, archaea; B, bacteria); fourth, size of the proteome in number of protein coding sequences. (57 KB DOC) [file pcbi.0010047.st002.doc]

Table S2

| **Accession number** | **Name** | **Superkingdom** | **Size** |
| --- | --- | --- | --- |
| NC_000964 | ***Bacillus subtilis*** | **B** | 8214 |
| NC_005363 | ***Bdellovibrio bacteriovorus*** | **B** | 7170 |
| NC_002927 | *Bordetella bronchiseptica* | **B** | 9993 |
| NC_001318 | ***Borrelia burgdorferi*** | **B** | 3279 |
| NC_004463 | ***Bradyrhizobium japonicum*** | **B** | 16669 |
| NC_003317 | ***Brucella melitensis*** | **B** | 6398 |
| NC_002528 | ***Buchnera aphidicola*** | **B** | 1148 |
| NC_005061 | ***Candidatus Blochmannia*** | **B** | 1295 |
| NC_002696 | ***Caulobacter vibrioides*** | **B** | 7476 |
| NC_002620 | ***Chlamydia muridarum*** | **B** | 3080 |
| NC_002932 | ***Chlorobaculum tepidum*** | **B** | 4504 |
| NC_005085 | ***Chromobacterium violaceum*** | **B** | 8814 |
| NC_001263 | ***Deinococcus radiodurans*** | **B** | 6299 |
| NC_000913 | ***Escherichia coli K-12*** | **B** | 9412 |
| NC_000907 | ***Haemophilus influenzae*** | **B** | 3410 |
| NC_000915 | ***Helicobacter pylori*** | **B** | 3155 |
| NC_002945 | ***Mycobacterium bovis*** | **B** | 7840 |
| NC_000962 | ***Mycoplasma tuberculosis*** | **B** | 8082 |
| NC_000908 | ***Mycoplasma genitalium*** | **B** | 964 |
| NC_003112 | ***Neisseria meningitidis*** | **B** | 4107 |
| NC_004193 | ***Oceanobacillus iheyensis*** | **B** | 6996 |
| NC_005042 | ***Prochlorococcus marinus*** | **B** | 3764 |
| NC_002516 | ***Pseudomonas aeruginosa*** | **B** | 11152 |
| NC_003198 | ***Salmonella enterica*** | **B** | 5129 |
| NC_004347 | ***Shewanella oneidensis*** | **B** | 9254 |
| NC_004741 | ***Shigella flexneri*** | **B** | 8136 |
| NC_002758 | ***Staphylococcus aureus*** | **B** | 6016 |
| NC_003098 | *Streptococcus pneumoniae* | **B** | 4439 |
| NC_004113 | ***Thermosynechococcus elongatus*** | **B** | 4989 |
| NC_000919 | ***Treponema pallidum*** | **B** | 2067 |
| NC_002162 | ***Ureaplasma urealyticum*** | **B** | 1225 |
| NC_002505 | ***Vibrio cholerae*** | **B** | 7663 |
| NC_005090 | ***Wolinella succinogenes*** | **B** | 2044 |
| NC_003919 | *Xanthomonas axonopodis* | **B** | 8854 |
| NC_002488 | *Xylella fastidiosa* | **B** | 5664 |
| NC­_004088 | ***Yersinia pestis*** | **B** | 8877 |
| NC_002607 | ***Halobacterium salinarum*** | **A** | 7667 |
| NC_003901 | ***Methanosarcina mazei*** | **A** | 6742 |
