# Supplementary material for: Entropic Stabilization of Proteins and Its Proteomic Consequences
Source: PLoS Comput Biol. 2005 Sep 30;1(4):e47. doi: 10.1371/journal.pcbi.0010047 (PMC1239905; doi:10.1371/journal.pcbi.0010047)
Supplement: Table S3 — Total of 12 genomes. Columns are as in Table S2. (33 KB DOC) [file pcbi.0010047.st003.doc]

Table S3

| **Accession number** | **Name** | **Domain of Life** | **Size** |
| --- | --- | --- | --- |
| **NC_000854** | ***Aeropyrum pernix*** | **A** | **4535** |
| **NC_000917** | ***Archaeoglobus fulgidus*** | **A** | **4829** |
| **NC_000909** | ***Methanococcus jannaschii*** | **A** | **3557** |
| **NC_003551** | ***Methanopyrus kandleri*** | **A** | **3374** |
| **NC_005213** | ***Nanoarchaeum equitans*** | **A** | **1072** |
| **NC_000868** | ***Pyrococcus abyssi*** | **A** | **1869** |
| **NC_003413** | ***Pyrococcus furiosus*** | **A** | **4202** |
| **NC_000961** | ***Pyrococcus horikoshii*** | **A** | **3690** |
| **NC_002754** | ***Sulfolobus solfataricus*** | **A** | **5971** |
| **NC_003106** | ***Sulfolobus tokodaii*** | **A** | **5653** |
| **NC_000918** | ***Aquifex aeolicus*** | **B** | **3122** |
| **NC_000853** | *Thermotoga maritima* | **B** | **3726** |
