# Supplementary material for: Entropic Stabilization of Proteins and Its Proteomic Consequences
Source: PLoS Comput Biol. 2005 Sep 30;1(4):e47. doi: 10.1371/journal.pcbi.0010047 (PMC1239905; doi:10.1371/journal.pcbi.0010047)
Supplement: Table S4 — Diff in σ, difference in number of standard deviation between respective expected and observed values. The null model used to calculate p-values represents random uncorrelated distribution of charged amino acids over proteomes resulting in binomial distribution for the content of each type of amino acids, from which p-values were calculated. (29 KB DOC) [file pcbi.0010047.st004.doc]

****Table S4****

| **Amino acid residue** | ***EC*(%)** | ***PF*(%)** | **Exp in *PF*** | **Obs in *PF*** | **Diff in σ**  **(p-value)** |
| --- | --- | --- | --- | --- | --- |
| **ARG** | 5.53 | 5.34 | 32469±525 | 31353 | 6.4(8·10^-11^) |
| **LYS** | 4.41 | 8.1 | 25893±471 | 47559 | 138(<10^-14^) |
| **ASP** | 5.1 | 4.36 | 29945±504 | 25600 | 26(<10^-14^) |
| **GLU** | 5.76 | 8.9 | 33820±534 | 52257 | 104(<10^-14^) |
